# The quantity-effect relationship and physiological mechanisms of different acupuncture manipulations on posterior circulation ischemia with vertigo: study protocol for a randomized controlled trial

**DOI:** 10.1186/s13063-015-0660-y

**Published:** 2015-04-11

**Authors:** Shi-Zhe Deng, Xiao-Feng Zhao, Ling-Hui Huang, Si He, Yan Wen, Chao Zhang, Guang Tian, Tian Wang, Fen-Fen Wu, Zhi-Hong Meng, Xue-Min Shi

**Affiliations:** Department of Acupuncture and Moxibustion, First Teaching Hospital of Tianjin University of Traditional Chinese Medicine, No. 314, West Anshan Avenue, 300193 Tianjin, China; Institute of Acupuncture and Moxibustion, First Teaching Hospital of Tianjin University of Traditional Chinese Medicine, No. 314, West Anshan Avenue, Tianjin, 300193 China

**Keywords:** Acupuncture, Quantity-effect relationship, Vertigo

## Abstract

**Background:**

Recent experiments have demonstrated that different needling manipulations may induce variable effects via diverse physiological mechanisms. A previous study indicated that needling at Fengchi (GB 20) improved cerebral blood flow in patients with vertigo induced by posterior circulation ischemia (PCI). In this study, we aim to explore the quantity-effect relationship and the physiological mechanisms underlying different acupuncture manipulations in PCI patients with vertigo.

**Methods/Design:**

We propose a pragmatic randomized and controlled trial. All participants, outcome assessors, and statisticians will be blinded. A total of 144 eligible participants will be randomized into one of four treatment groups receiving acupuncture at Fengchi (GB 20) with different one-minute manipulation parameters. Group 1 will receive twirling at a frequency of 60 times per minute toward the contralateral outer canthus at a depth of 0.5 to 0.8 cun. Group 2 will receive twirling at a frequency of 60 times per minute toward the Adam’s apple at a depth of 0.5 to 0.8 cun. Group 3 will receive twirling at a frequency of 120 times per minute toward the contralateral outer canthus at a depth of 0.5 to 0.8 cun. Group 4 will receive twirling at a frequency of120 times per minute toward the Adam’s apple at a depth of 0.5 to 0.8 cun. Additional points will be added based on individualized pattern diagnoses. The participants will receive 14 acupuncture sessions over 3 to 4 weeks. The subjects will be assessed at two time points: baseline and post-treatment. The primary outcome measurements will include subjective measurements (Vertebrobasilar System Ischemic Neurological Impairment Scale, UCLA Dizziness Questionnaire, Activities of Daily Living Scale, and Psychological and Social Adaptation Scale) and objective measurements (Transcranial Doppler, carotid ultrasonography and changes in cerebral oxygenation) to reduce bias arising from the placebo effect. We will use metabolomics to investigate the mechanisms underlying the different manipulation parameters.

**Discussion:**

This trial aims to explore the quantity-effect relationship between different acupuncture manipulations and their clinical effects. The results from this study may help explain the contradictory results found in acupuncture studies that practice different manipulations.

**Trial registration:**

Chinese Clinical Trial Registry: ChiCTR-RTRCC-12002675 (registered on 14 November 2012).

## Background

Vertigo is the main symptom of posterior circulation ischemia (PCI) [1]. Patients with vertigo resulting from posterior circulation ischemia (PCIV) are mainly middle-aged or older, and most patients are male [2]. The most common causes of PCI are atherosclerotic lesions, embolism, and small penetrating arterial disease, which lead to tissue function defects of the inner ear, brainstem, and cerebellum [2-5].

Traditional Chinese medicine (TCM) theorizes that vertigo is caused by a loss of nourishment in the upper orifices resulting from pathogenic wind, heat, phlegm, deficiency, or stasis. Vertigo is thought to be closely related to the liver, kidney, heart, and spleen [6]. Fengchi (GB 20) is one of the main acupoints used to treat vertigo, and acupuncture at Fengchi (GB 20) promotes the flow of qi and blood, pacifies the liver, and normalizes bile secretion [7]. Our previous study found that needling at Fengchi (GB 20) could improve cerebral blood flow in patients with PCIV [8].

Recent human and animal experiments have indicated that different needling manipulations could result in different effects through different physiological mechanisms [9,10]. However, few studies have investigated the influence of different needling directions and frequencies and their inherent mechanism of action.

We hypothesize that an ideal quantity of stimulation applied at Fengchi (GB 20) may induce an optimal effect in PCIV patients. This hypothesis is based on the quantity theory of acupuncture manipulation developed by academician Shi Xuemin, who stated, ‘acupuncture is an intervention for physical stimulation; so the quantity of stimulation including the intensity, frequency, duration, direction of the needle tip, and interval between intervention sessions may play a vital role in clinical outcomes. Just as with drugs, the effect of acupuncture is derived from the quantity of intervention. The optimal effect of acupuncture depends on the optimal stimulation quantity. Therefore, acupuncture intervention should be clearly quantified in clinical acupuncture research.’ In this study, we aim to explore whether different needling manipulations (direction and frequency) at Fengchi (GB 20) can influence the clinical effects of acupuncture via different physiological mechanisms. We also aim to investigate the most effective acupuncture parameters in patients with PCIV.

## Methods/Design

### Study design

This trial is a pragmatic randomized and controlled study. A previous study conducted in our hospital demonstrated that needling at Fengchi (GB 20) was effective for the treatment of PCIV [8], but the optimal treatment parameters are unknown. This study protocol was approved by the regional ethics review board of Tianjin University of Traditional Chinese Medicine (approval number: TJUTCM-EC20110005). This trial is registered in the Chinese Clinical Trial Registry under the following identifier: ChiCTR-RTRCC-12002675. This trial will be performed in accordance with the principles of the Declaration of Helsinki (Version Edinburgh 2000). Informed consent will be obtained from each participant before enrolment. This work has not been published elsewhere.

### Randomization

Patients will be consecutively and randomly assigned to one of four groups in a 1:1:1:1 ratio. Randomization will occur before the first acupuncture treatment. A computerized random number generator will be used to generate the randomization sequence. Allocation concealment will be performed via sequentially numbered opaque, sealed envelopes by an investigator who is not involved in recruitment or assessment of the results.

### Blinding

The patients, outcome assessors, the statistician, data-handling personnel, transcranial Doppler operators, and carotid ultrasonography and metabolomics operators will be blinded during the study. However, the acupuncture practitioners will not be blinded. An inquiry will be performed to investigate whether the subjects were blinded to the treatment that they received.

### Acupuncture groups

We aim to explore the quantity-effect relationship among various acupuncture parameters rather than to demonstrate the effectiveness of Fengchi (GB 20) needling. Therefore, a sham control group will not be used. We will use a pragmatic design to directly compare four parameters.

### Recruitment strategies

This trial was created by the Ministry of Science and Technology of the People’s Republic of China and will be administered by the State Administration of Traditional Chinese Medicine of the People’s Republic of China. The study will be performed at the First Teaching Hospital of Tianjin University of TCM, which is named the National Clinical Research Centre of Acupuncture and Moxibustion. We will place notices on printed recruitment posters and electronic bulletin boards. Interested patients will be referred to specific researchers and will receive screening evaluations. Imaging diagnoses from Transcranial Doppler (TCD) will be used in this study. The subjects will receive a routine blood examination, routine urine and feces tests, liver and kidney function tests, and an electrocardiogram (ECG) before recruitment.

All patients who meet the terms listed above will be given time to decide whether to participate in the study. The patients must fully understand all details of the informed consent before signing. Patients satisfying the diagnostic criteria will be introduced to the acupuncturist.

### Diagnostic criteria

The diagnostic criteria for PCIV are in accordance with the WHO criteria [11]. Patients must experience dizziness or vertigo that may be accompanied by the following: acute unsteadiness or ataxia; unilateral or bilateral (or involving one side after the other) visual, motor or sensory disturbances; double vision; dysarthria or swallowing impairment; acute impairment of consciousness; or acute confusion. Patients must have TCD or magnetic resonance imaging results that indicate vertebrobasilar insufficiency.

### Eligibility

#### Inclusion criteria

Participants will be included if they meet all of the following conditions:

1. Diagnosis of vertebrobasilar insufficiency.
2. Aged between 40 and 75 years.
3. Volunteered to participate in the acupuncture treatment and can cooperate with the treatment.
4. Provide signed informed consent.

#### Exclusion criteria

Participants will be excluded if they meet any of the following conditions:

1. Cerebral hemorrhage or bulbar paralysis.
2. Aural or ocular vertigo.
3. Systolic blood pressure greater than 180 mmHg and/or diastolic blood pressure greater than 120 mmHg.
4. Glasgow Coma Scale score of less than 15 or Hasegawa’s Dementia Scale score of less than 27.5.
5. Serious heart, liver, or kidney diseases, critical illnesses that require operation, pregnancy, lactation, or serious dysphrenia.
6. Participation in another current clinical trial or participation in a clinical trial within 3 months of recruitment.

### Interventions

The interventions were designed by Professor Shi Xuemin, an academician at the Chinese Academy of Engineering, who promotes the standardization of acupuncture manipulation.

Fengchi (GB 20) is located on the posterior neck, inferior to the occipital bone, in the depression between the origins of sternocleidomastoid and the trapezius muscles [12]. Additional points will be added based on individualized pattern diagnoses.

### Rationale for acupoint selection and different manipulations of Fengchi (GB 20)

According to TCM theory, Fengchi (GB 20) is one of the main acupoints that can be used to help treat vertigo. Studies have demonstrated that acupuncture at Fengchi (GB 20) can affect cerebral blood flow [8,13].

Based on the quantity theory of acupuncture manipulation [14], differences in manipulations at Fengchi (GB 20) may induce different clinical effects. We selected different directions (the contralateral outer canthus or the Adam’s apple) and different frequencies (60 times per minute or 120 times per minute) to investigate our hypothesis. Needling manipulations will be performed manually by one experienced (more than 20 years) acupuncturist. Before conducting this trial, the acupuncturist received professional training to ensure the accuracy of needling replication.

The four manipulations used in this study are often used in clinical practice. Some studies state that the direction of acupuncture at Fengchi (GB 20) should be toward the contralateral outer canthus [15]. However, other studies acupuncture at Fengchi (GB 20) toward the Adam’s apple [16]. The two frequencies were chosen based on clinical experience.

### Protocol for acupuncture manipulation

**Figure 1:**
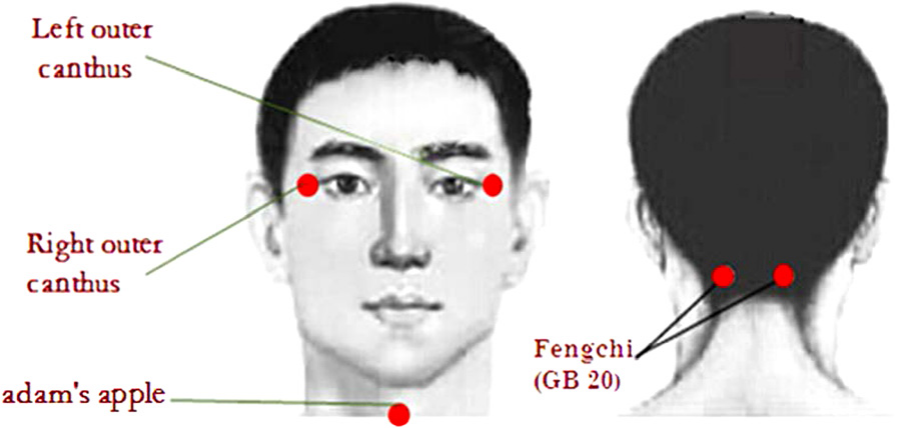
Directions of acupuncture interventions. When acupuncture is initiated at the left Fengchi (GB 20), it will proceed toward the right outer canthus or the Adam’s apple. When acupuncture is initiated at the right Fengchi (GB 20), it will proceed toward the left outer canthus or the Adam’s apple.

Sterile disposable stainless steel needles (length: 40 mm, diameter: 0.25 mm; Hwatuo, Suzhou Medical Supplies Factory Co., Ltd., Suzhou, China) will be used on patients. For the main acupoint, Fengchi (GB 20), each group will be needled with twisting for a minute. Group 1 will receive twirling with a frequency of 60 times per minute toward the contralateral outer canthus at a depth of 0.5 to 0.8 cun. Group 2 will receive twirling with a frequency of 60 times per minute toward the Adam’s apple at a depth of 0.5 to 0.8 cun. Group 3 will receive twirling with a frequency of 120 times per minute toward the contralateral outer canthus at a depth of 0.5 to 0.8 cun. Group 4 will receive twirling with a frequency of 120 times per minute toward the Adam’s apple at a depth of 0.5 to 0.8 cun (Figure 1). Additional points will be added based on individualized pattern diagnoses. For the retention of phlegm-dampness, we will add Fenglong (ST40), Zusanli (ST36), and Yinlingquan (SP9). For hyperactivity of Liver Yang, we will add Taichong (LR3) and Taixi (KI3). For deficiency of Kidney Yin, we will add Taixi (KI3), Zhaohai (KI6), and Neiguan (PC6). For blood stasis, we will add Hegu (LI4), Sanyinjiao (SP6), Yinxi (HT6), and Xuehai (SP10). For qi and blood deficiency, we will add Qihai (CV6), Zhongwan (CV12), Zusanli (ST36), and Laogong (PC8). After *de qi*, the needles will be retained for 20 minutes. Other interventions, such as moxibustion, cupping, and herbs will not be used. The participants will receive a total of 14 sessions over 3 to 4 weeks (3 to 4 sessions per week).

### Outcome measurement

Outcome measurements will be assessed at baseline and after the treatment phase. We will use multiple outcome measurements, including subjective and objective parameters.

The patients will be required to complete several subjective questionnaires before and after treatment, including the following: the Vertebrobasilar System Ischemic Neurological Impairment Scale (VBS-INIS) (range: 0 to 22) [17], UCLA Dizziness Questionnaire (UCLA-DQ) (range: 5 to 25) [18], Activities of Daily Living Scale (ADL) (range: 14 to 56) [19], and Psychological and Social Adaptation Scale (range: 0 to 20) [20]. All scales have been validated in previous studies.

The VBS-INIS assesses the degree of neurological impairment caused by vertebrobasilar ischemia. Common signs and symptoms include dizziness, deafness or tinnitus, double vision, dysarthria, or swallowing impairment. The scale was demonstrated to be uniform, stable, and reliable. Higher scores indicate more severe damage.

The UCLA-DQ is used to measure the severity and frequency of dizziness. The questionnaire contains five items to evaluate the patients in terms of daily activity, quality of life, and their fear of dizziness.

The ADL is a validated questionnaire that has been widely used to assess ability of an individual to function in daily life. The questionnaire consists of two parts: the Physical Self-Maintenance Scale and the Instrumental Activities of Daily Living Scale.

The Psychological and Social Adaptation Scale mainly describes the impact of dizziness on daily life and work. The scale assesses psychological parameters, such as temper, worry, fear, quality of sleep, and communication with others. The reliability, availability, and sensitivity of this scale had been clinically tested [20].

**Figure 2:**
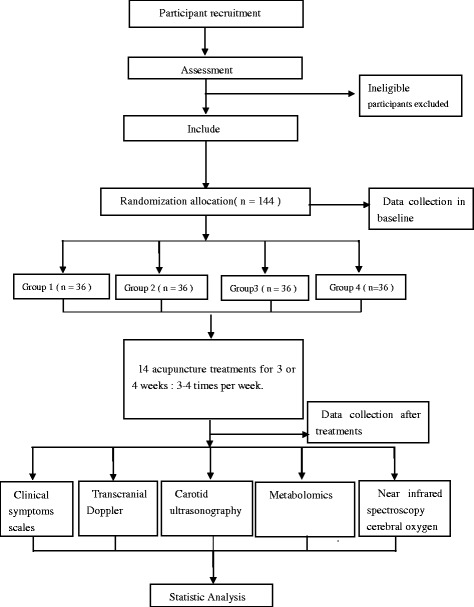
Study sequence. A total of 144 eligible participants will be randomized into one of four treatment groups receiving acupuncture for one minute with different manipulation parameters. Group 1 will receive twirling with a frequency of 60 times per minute toward the contralateral outer canthus at a depth of 0.5 to 0.8 cun. Group 2 will receive twirling with a frequency of 60 times per minute toward the Adam’s apple at a depth of 0.5 to 0.8 cun. Group 3 will receive twirling with a frequency of 120 times per minute toward the contralateral outer canthus at a depth of 0.5 to 0.8 cun. Group 4 will receive twirling with a frequency of 120 times per minute toward the Adam’s apple at a depth of 0.5 to 0.8 cun. The participants will receive 14 acupuncture sessions over 3 to 4 weeks. The subjects will be assessed at two time points: baseline and post-treatment. The primary outcome measurements will include subjective measurements (Vertebrobasilar System Ischemic Neurological Impairment Scale, UCLA Dizziness Questionnaire, Activities of Daily Living Scale, and Psychological and Social Adaptation Scale) and objective measurements (Transcranial Doppler, carotid ultrasonography, and changes in cerebral oxygenation) to reduce bias arising from the placebo effect. We will use metabolomics to investigate the mechanisms underlying the effects of the different manipulation parameters.

Objective parameters will also be used to compare differences in cerebral blood flow via TCD and carotid ultrasonography. Changes in cerebral oxygenation will be investigated using near-infrared spectroscopy, and the inherent mechanism underlying acupuncture treatment efficacy will be investigated using metabolomics (Figure 2).

Certain medical assessments, such as general information, vital signs, symptoms, physical signs, previous history, complications, and information from the TCM diagnosis, will also be recorded.

### Patient safety

Patient safety will be assessed using laboratory tests (for example, routine blood, urine and feces tests, and hepatic and renal function) and electrocardiography before and after treatment. These tests will also be used to identify potential side effects. For every session of acupuncture, curative effects, duration, syndrome, adverse reactions or events (for example, bleeding, hematoma, or serious pain), and patient dropout will be recorded. If any patients experience adverse events, follow-up monitoring will be performed until the adverse events resolve.

### Quality control

Different factors have been considered to reduce bias. All researchers are trained to diagnose posterior circulation ischemia, to observe and record data, and to fill in the case report forms. During the trial, supervisors will regularly audit the trial to verify the consistency of the raw data and the recorded data. The data analyst will be involved early on in the study, so that the entire trial can be monitored.

### Statistical analysis

#### Sample size

We estimated the sample size using a formula, as was performed in relevant literature studies. We plan to enroll a total of 144 participants, with 36 individuals in each group, allowing for a 20% withdrawal rate.

#### Analysis strategy

Statistical analysis will be performed using the statistical software package SPSS 16.0 for Windows (SPSS Inc., Chicago, IL, USA). Differences between the baseline characteristics of each treatment group will be analyzed using an analysis of variance (ANOVA) for continuous variables such as age, body mass index, and disease course. Comparisons among the baseline characteristics of the four groups for categorical variables, such as gender and medical history, will be made using the chi-square test. Paired sample *t*-tests will be used to compare the subjective and objective outcome measurements before and after treatment. Comparisons of the subjective and objective outcome measurements among the four groups will be performed by ANOVA. A *P* value of less than 0.05 will be considered statistically significant. Qualified statisticians will perform the statistical analyses in a blinded manner.

## Discussion

Typically, acupuncture manipulations refer to the various manipulations performed by an acupuncturist after the needle is inserted. TCM theory suggests that various styles of acupuncture manipulations are closely associated with different clinical effects. Long-term practices in our hospital (The National Clinical Research Centre of Acupuncture and Moxibustion) also support this theory. Recent studies [9,10,21] have demonstrated that different acupuncture manipulations exert different influences on pain, acupuncture sensation (*de qi*), and blood perfusion. However, few studies have investigated the direct relationship between needle manipulations and clinical outcomes. Therefore, this trial aims to investigate the potential clinical differences that result from diverse manipulations.

Rigid quality control will be used in this trial to reduce potential bias. Experienced subjects might be able to distinguish real acupuncture from sham acupuncture in appearance or sensation [22]. Therefore, sham acupuncture might not be effective in the Chinese population. All groups will receive verum acupuncture, and patients will have their backs turned to the acupuncturist. The subjects will not observe the manipulations conducted by the acupuncturist. Moreover, an inquiry will be performed to investigate whether the subjects were truly blinded to the treatment received.

We selected a pragmatic randomized controlled trial after an extensive discussion. Although both pragmatic and explanatory randomized controlled trials play important roles in the evaluation of health care interventions, the objectives and procedures of the two methods are different [23]. The primary objective of the explanatory trial is to determine whether acupuncture is better than placebo. Explanatory trials are an absolute proof for the validation of a therapeutic intervention [24]. To evaluate complex interventions such as acupuncture, the explanatory randomized controlled trial might not be an appropriate study design [25]. Instead, pragmatic randomized controlled trials can effectively investigate the overall impact of a group of treatments. Pragmatic trials can also provide a real comparison in clinical practice, which can provide a cost-effective analysis and can directly relate to policy. In pragmatic trials, the important question is whether the overall effect is worth the cost [23,26,27]. From a methodological perspective, explanatory RCTs demand rigorously controlled experimental conditions and the use of a placebo control [28]. Using a less rigidly controlled study design than those of explanatory RCTs, pragmatic trials can compare different interventions under actual clinical conditions, and placebo controls are not required [29]. However, pragmatic trials contain inherent deficiencies. Because of differences in needling techniques, treatments often include subtle variations, rendering the trials difficult to replicate [30]. To overcome this flaw, we will use a standardized acupuncture manipulation. The acupuncture treatments will be conducted by one acupuncturist with adequate clinical experience. Before conducting the trial, the acupuncturist received professional training to ensure accurate needle operation. Therefore, this trial should demonstrate adequate reproducibility.

This study is based on the quantity theory of acupuncture manipulation developed by Shi Xuemin. This method aims to define and quantify acupuncture manipulation. The quantity theory of acupuncture manipulation has rendered the quantification of acupuncture more feasible and has contributed to the standardization, repeatability, and control of acupuncture studies. This method could be used to determine the optimal treatment method based on different parameters.

This study includes some limitations that may affect the results. First, we will not conduct a follow-up study. We consider the observation of the short-term therapeutic clinical effects sufficient to investigate our hypothesis, but our study design is insufficient for evaluating long-term effects. Moreover, the outcome measurement scales may contain bias when translated into Chinese, which could influence the results of the trial. Furthermore, because Chinese patients expect acupuncture to be effective, the placebo effect may exert a stronger influence on the results than if the study is performed with mostly Western patients. To reduce this bias, we will use multiple outcome measurements, including subjective and objective outcome measurements. The placebo effect might significantly influence subjective outcomes but should not significantly affect objective measurements [31]. Finally, we did not use sham acupuncture. Because Chinese patients generally trust acupuncture, informed consent for the use of sham acupuncture is difficult to obtain [32]. A previous study also demonstrated that acupuncture is an effective intervention for PCIV; therefore, we want to compare different acupuncture methods [8].

The results of this study may help explain the contradictory results obtained from acupuncture studies if the different manipulations can induce different treatment effects [33]. This study could also elucidate the overall treatment effect of acupuncture on patients with PCIV. The use of both subjective and objective outcome measurements should allow the identification of significant differences between these two outcome measurements.

In conclusion, this study will attempt to explore the relative efficacies of different acupuncture manipulations and their mechanisms of action. Because the focus of this study is highly specific, the findings may have implications for a number of ongoing debates in the field of acupuncture research.

### Trial status

The trial had finished the recruitment phas in August 2014.We will report the results in later article.

## Abbreviations

ADL: Activities of daily living
CRF: Case report form
DBP: Diastolic blood pressure
ECG: Electrocardiogram
MRI: Magnetic resonance imaging
PCIV: Vertigo resulting from posterior circulation ischemia
SBP: Systolic blood pressure
TCD: Transcranial doppler
UCLA-DQ: University of California, Los Angeles-dizziness questionnaire
VBI: Vertebrobasilar insufficiency
VBS-INIS: Vertebrobasilar system ischemic neurological impairment scale

## References

[1] Searls DE, Pazdera L, Korbel E, Vysata O, Caplan LR (2012). Symptoms and signs of posterior circulation ischemia in the new England medical center posterior circulation registry. Arch Neurol.

[2] Caplan LR, Wityk RJ, Glass TA, Tapia J, Pazdera L, Chang HM (2004). New England medical center posterior circulation registry. Ann Neurol.

[3] Savitz SI, Caplan LR (2005). Vertebrobasilar disease. N Engl J Med.

[4] Vuilleumier P, Bogousslavsky J, Regli F (1995). Infarction of the lower brainstem. Clinical, aetiological and MRI-topographical correlations. Brain.

[5] Caplan L (2000). Posterior circulation ischemia: then, now, and tomorrow. The Thomas Willis Lecture-2000. Stroke.

[6] Cheng XN (2012). Chinese Acupuncture and Moxibustion.

[7] Chen S (2003). Clinical application of Fengchi (GB 20) Point. J Tradit Chin Med.

[8] Zhao HX, Wang WZ, Wang S, Zhou P (1997). Effect of acupuncture in patient with vertebrobasilar insufficiency, reports of 102 cases. Zhongguo Zhen Jiu.

[9] Choi YJ, Lee JE, Moon WK, Cho SH (2013). Does the effect of acupuncture depend on needling sensation and manipulation?. Complement Ther Med.

[10] Langevin HM, Bouffard NA, Churchill DL, Badger GJ (2007). Connective tissue fibroblast response to acupuncture: dose-dependent effect of bidirectional needle rotation. J Altern Complement Med.

[11] Goldstein M, Barnett HJM, Orgogozo JM, Sartorius N, Symon L, Vereshehagin NV (1989). Stroke - 1989. Recommendations on stroke prevention, diagnosis, and therapy report of the WHO task force on stroke and other cerebrovascular disorders. Stroke.

[12] World Health Organization. WHO standard acupuncture point locations in the Western Pacific Region. Beijing. 2010.

[13] Yuan XJ, Hao XS, Lai ZP, Zhao H, Liu WY (1996). Influence of acupuncturing Fengchi (GB 20) point on cerebral blood flowing. J Tradit Chin Med.

[14] Bian JL, Zhang CH (2003). Conception and core of academician Shi Xuemin’s acupuncture manipulation quantitative arts. Zhongguo Zhen Jiu.

[15] Luo YF (1996). Subject of acupoint.

[16] Shi XM, Yang ZG, Zhou JZ, Han JX, Zhang CS, Bian JL (1999). Clincal and experimental studies on acupuncture treatment for 325 cases of pseudobulbar paralysis. Zhongguo Zhen Jiu.

[17] Ji RJ, Jia JP, Meng R, Xu Y, Zhou P, Ma X (2008). Establishment and reliability, validity testing of the Vertebrobasilar system ischemic neurological impairment scale. J Brain Nerv Dis.

[18] Honrubia V, Bell TS, Harris MR, Baloh RW, Fisher LM (1996). Quantitative evaluation of dizziness characteristics and impact on quality of life. Am J Otol.

[19] Lawton MP, Brody EM (1969). Assessment of older people: self-maintaining and instrumental activities of daily living. Gerontologist.

[20] Wang CH, Zhuo DH (1998). A preliminary evaluation of symptoms and functions of patients with cervical vertigo. Chin J Rehabil Med.

[21] Lin JG, Chen CH, Huang YC, Chen YH (2012). How to design the control group in randomized controlled trials of acupuncture?. Evid Based Complement Alternat Med.

[22] Tsukayama H, Yamashita H, Kimura T, Otsuki K (2006). Factors that influence the applicability of sham needle in acupuncture trials two randomized, single-blind, crossover trials with acupuncture-experienced subjects. Clin J Pain.

[23] MacPherson H (2004). Pragmatic clinical trials. Complement Ther Med.

[24] Helms PJ (2002). ‘Real world’ pragmatic clinical trials: what are they and what do they tell us?. Pediatr Allergy Immunol.

[25] Fitter MJ, Thomas KJ (1997). Evaluating complementary therapies for use in the NHS: ‘horses for courses’. Part 1. The design challenge. Complement Ther Med.

[26] MacPherson H, Bland M, Bloor K, Cox H, Geddes D, Kang’ombe A (2010). Acupuncture for irritable bowel syndrome: a protocol for a pragmatic randomised controlled trial. BMC Gastroenterol.

[27] Maningat P, Breslow JL (2011). Needed: pragmatic clinical trials for statin-intolerant patients. N Engl J Med.

[28] Cowan D (2011). Methodological issues in evaluating auricular acupuncture therapy for problems arising from the use of drugs and alcohol. Acupunct Med.

[29] Kaptchuk TJ, Chen KJ, Song J (2010). Recent clinical trials of acupuncture in the west: responses from practitioners. Chin J Integr Med.

[30] Kim EJ, Lim CY, Lee EY, Lee SD, Kim KS (2013). Comparing the effects of individualized, standard, sham and no acupuncture in the treatment of knee osteoarthritis: a multicenter randomized controlled trial. Trials.

[31] Wechsler ME, Kelley JM, Boyd IO, Dutile S, Marigowda G, Kirsch I (2011). Active albuterol or placebo, sham acupuncture, or no intervention in asthma. N Engl J Med.

[32] Xu SB, Huang B, Zhang CY, Du P, Yuan Q, Bi GJ (2013). Effectiveness of strengthened stimulation during acupuncture for the treatment of Bell palsy: a randomized controlled trial. CMAJ.

[33] Ernst E (2009). Complementary and alternative medicine: between evidence and absurdity. Perspect Biol Med.

